# The Liquefaction of Gases

**Published:** 1878-06

**Authors:** 


### ARTICLE II.

### *The Liquefaction of Gases Hitherto Thought to be Incoercible.*

Towards the end of December considerable interest was excited by the announcement that the liquefaction of oxygen had been effected at Geneva by M. Raoul Pictet, on the 22d of December. When, however, the subject came before the Academy of Sciences, at its *seance* on the 24th, the interest was heightened by the announcement that M. Pictet had been to some extent anticipated, in that M. Cailletet, who a short time since succeeded in liquefying binoxide of nitrogen, had also effected the liquefaction of oxygen and of carbonic acid on the 2d of December, although in consequence of some consideration as to his pending election to the body, instead of the fact being published at once, a record of it had been intrusted in a sealed packet to the custody of the Secretary to the Academy on the 3d. At the sitting of the Academy on the 31st December it was announced that M. Cailletet had succeeded in liquefying nitrogen, hydrogen and atmospheric air. The following account of the operation by which these results were effected is taken from *Comptes Rendus* of December 24 and 31.

To give M. Cailletet precedence. The following are the terms in which M. Cailletet communicated his success with oxygen and carbonic oxide to his old master, M. Sainte-Claire Deville, and which formed the basis of the sealed packet:

"I am anxious to tell you first and without the loss of a moment that I have to-day liquefied carbonic oxide and oxygen. I am, perhaps, wrong to say liquefy, for at the temperature obtained by the evaporation of sulphurous acid, *i. e.*, —29° and 300 atmospheres, I do not see the liquid, but a fog so thick that I may conclude it to be a vapor very near the point of liquefaction. I am writing to-day to M. Deleuil for protoxide of nitrogen, by the help of which I

shall, no noubt, be able to watch the flow of a stream of carbonic oxide and oxygen.

"P. S. I have just made an experiment that puts my mind at rest. I compressed hydrogen at 300 atmospheres, and after cooling at —28°, I allowed it to expand suddenly. There was no trace of fog in the tube. My gases (CO and O) are, therefore, nearly upon liquefaction, this fog being produced only with vapors near liquefying. M. Bethelot's anticipations are, consequently, being completely realized."

The following is the more detailed account read before the Academy on the 24th of December:

"If oxygen or pure carbonic oxide be inclosed in a tube of the form I have previously described, and placed in the compression apparatus already exhibited before the Academy,* and the gas be brought down to a temperature of —29° by means of sulphurous acid at the pressure of about 300 atmospheres, these two gases retain their gaseos condition. But if they are suddenly allowed to expand, so as to produce, according to Poisso's law, a temperature of at least 200° below the initial temperature, immediately an intense fog, produced by the liquefaction, and, perhaps, solidification of the oxygen or carbonic oxide, becomes perceptible.

"This phenomenon is observed also on the expansion of carbonic acid and the protoxide and binoxide of nitrogen when under strong pressure.

"This fog is produced with oxygen even when the gas is at the common temperature, provided time is given for the escape of heat it acquires by the mere act of compression.

"This I demonstrated by experiments conducted on Sunday, December 16th, at the chemical laboratory of the Ecole Normal Superieure, before a number of scientific men, among whom were some members of the Academy of Sciences. I had hoped to find at Paris, together with the

* This compression apparatus, which is described in the *Comptes Rendus* for November 5 last, is a hollow steel cylinder, in which the gas is compressed by means of a hydraulic pump with the intervention of a layer of mercury.

materials necessary for the production of a high degree of cold (protoxide of nitrogen or liquid carbonic acid,) a pump capable of taking the place of my compression apparatus at Chatillon-sur-Seine. Unfortunately, a pump well fixed and suited to this kind of experiment could not be found at Paris, and I was obliged to send to Chatillon-sur-Seine for the refrigerating material for collecting the condensed matters on the walls of the tube.

"To know whether oxygen and carbonic oxide are in a liquid or solid form in the fog would require an optical experiment more easy to imagine than to carry out, on account of the form and thickness of the tubes containing them. Chemical reactions will also allow of the demonstration that the oxygen is not transformed into ozone in the act of compression. These questions I shall endeavor to solve with the help of the apparatus which I am now having made.

"Under the same conditions of temperature and pressure even the most rapid expansion of pure hydrogen gives no trace of nebulous matter. There remains, therefore, only nitrogen to study, and the small solubility of this gas in water leads me to suppose that it will be very refractory to any change of condition."

At the meeting of the following week, however, M. Cailletet was able to report a great advance towards the solution of this problem in the following "Note on the Condensation of Reputedly Incoercible Gases:"

"I have continued my experiments upon the liquefaction of gases, and I am happy to announce that I have succeeded in liquefying nitrogen atmospheric air. Hydrogen itself has given indications of liquefaction, as will presently appear.

"The following are some details of my experiments:

"*Nitrogen*—Pure and dry nitrogen, submitted to a pressure of about 200 atmospheres, at a temperature of—13°, and the pressure then suddenly relaxed, is condensed in the clearest manner. There is produced at first a matter similar to an atomized liquid (*liquide pulverise,*) in particles of an appreciable volume; then the liquid gradually disappears from the sides towards the center of the tube, forming at last a sort of vertical column directed along the axis of the tube itself. The total duration of the phenomenon is about three seconds.

"These appearances leave no doubt of the true character of the phenomenon. I first made the experiment at home, at the temperature of—29°, and I repeated it yesterday a great number of times in the laboratory of the Normal School, in the presence of several scientific men and members of the Academy, among whom I may mention, with his consent, the venerated M. Boussingault.

"*Hydrogen*—Hydrogen has always been regarded as the most incoercible gas, because of its slight density and the nearly complete conformity of its mechanical properties with those of a perfect gas. It was therefore only with extreme distrust as to the result that I decided to submit it to the same tests as have determined the liquefaction of the other gases.

"In my first attempts, I could not recognize anything unusual, but, as it often happens in experimental science, the habit of observing the phenomenon leads to being able to recognize signs under conditions where at first they passed unperceived. This is what occurred with hydrogen. In an experiment to-day, in the presence of MM. Barthelot, H. Sainte-Claire Deville and Mascart, who have kindly authorized me to invoke their testimony, I have succeeded in observing indications of the liquefaction of hydrogen, in conditions of evidence which did not appear doubtful to any of the learned men who witnessed the experiment. This has been repeated a great number of times. In operating with pure hydrogen, submitted to a pressure of about 280 atmospheres, and then suddenly released, we have observed the formation of an excessively fine and subtle fog suspended through the length of the tube, and which disappeared suddenly. The appearance of this fog, notwithstanding its extreme subtlety, has appeared incontestable to all the scientific men who have witnessed the experiment to-day, and they have taken care to repeat the experiment several times so as not to allow of any doubt as to its reality.

"*Air*—Having liquefied oxygen and nitrogen, the liquefaction of air had been thus demonstrated; however, it appears to me to be interesting to make the direct experiment, and, as might be expected, it has succeeded perfectly. It is unnecessary to say that the air had been previously dried and deprived of carbonic acid. Thus has been confirmed the exactitude of the views put forward by the founder of modern chemistry, M. Lavoisier, upon the possibility of making air assume the liquid state, producing matters endowed with new and unknown properties."

The object at which I have been aiming for three years past is to demonstrate experimentally that molecular cohesion is a general property of bodies, without any exception. If the permanent gases cannot be liquefied, it would be necessary to conclude that their constituent particles do not attract each other, and are thus exempt from this law. To succeed by experimental means in bringing the particles of a gas into the closest possible proximity, and thus accomplish its liquefaction, certain indispensable conditions have to be complied with, which I summarize as follows: (1) To have an absolutely pure gas, free from any trace of a foreign gas; (2) to have at command very powerful means of compression; (3) to obtain an intense degree of cold, and the abstraction of heat at these low temperatures; (4) to have a large surface of condensation maintained at these low temperatures; (5) to be able to utilize the expansion of the gas under considerable pressure to the atmospheric pressure, which expansion, added to the preceding means, compels liquefaction. These five conditions being fulfilled, we may formulate the following problem: When a gas is compressed at 500 or 600 atmospheres, and kept at a tempera ture of—100° or—140° and then allowed to expand to the pressure of the atmosphere, one of two things must happen: either the gas, obeying the action of cohesion, becomes liquid, giving up its heat of condensation to the portion of the gas, which expands and is lost to the gaseous form; or, under the assumption that cohesion is not a general law, the gas passes beyond absolute zero, that is, becomes inert, a dust without consistence. The work of expansion would be impossible, and the loss of heat absolute.

"Impressed with the certainty that thermo-dynamic equations rest upon fixed numerical forms, I sought to contrive some mechanical means for realizing the conditions summarized above, and selected a complex apparatus that I may describe as follows:

"I took two suction force pumps such as I use industrially in my ice-making apparatus. These I coupled so that the suction of the one corresponds to the compression of the other. The suction in the first communicates with a tube 1.10 m. long, filled with liquid sulphurous acid. Under the influence of a perfect vacuum, the temperature of this liquid is rapidly lowered to—65° or even—73°, the extreme limit which has been obtained. Into this tube of sulphurous acid there passes a second smaller tube, measuring six centimetres in exterior diameter, and of the same length as the tube in which it is inclosed. These two tubes have a common bottom. In the central tube I compressed carbonic acid produced by the decomposition of Carrara marble with hydrochloric acid. This gas was dried, then collected under an oil-gas holder of one cubic metre capacity. With a pressure varying between four and six atmospheres carbonic acid is easily liquefied under these conditions. The fluid flows down by its own weight into a copper tube four metres in length and four centimetres in diameter.

"Two pumps, coupled in the same way as the first pair, volatilize the carbonic acid in the gasometer, and the long tubes fill with the liquid acid. Admission to the pumps is regulated by a three-way tap; a screw-regulating tap intercepts, if desired, the entry of liquid carbonic acid into the long tube; it is placed between the carbonic acid condenser and this long tube. When the regulating tap is closed, and the two pumps suck up the vapor from the liquid carbonic acid contained in the four-metre tube, the lowest temperature is produced that can be got; the carbonic acid solidifies, its temperature falling to about—140°. The work of abstracting heat is kept up by the pumps, which have a joint cylinder capacity of three litres per stroke and make 100 strokes per minute. Both the sulphurous acid tube and the carbonic acid tube are inclosed in sawdust and baize to prevent radiation.

" Into the interior of the carbonic acid tube there passes a fourth tube, five metres in length, fourteen millimetres in external diameter and four millimetres in internal diameter, which serves as the oxygen compresser. This long tube is surrounded by solid carbonic acid, and the whole of its surface is brought to the lowest temperature that it is possible to obtain. These two long tubes are connected at the lower ends with the carbonic acid tube; consequently, the small tube goes beyond the other by about one metre. This portion I bend towards the ground, giving both tubes a slightly inclined position. The small central tube is bent down, and screwed into the neck of a strong cylindro-conical retort, made of wrought iron, the walls of which are 35 millimetres thick. Its height is 28 centimetres and diameter 17 centimetres. This retort contains 700 grammes of potassium chlorate and 256 grammes of potassium chloride mixed together, fused, pulverized, and placed in the retort perfectly dry. I heat the retort when the double circulations of sulphurous acid and carbonic acid have lowered the temperature to the point desired. The chlorate of potassium is decomposed slowly at first, but tolerably rapidly at the end of the operation. A pressure gauge at the end of the long tube enables me to watch the pressure and the progress of the reaction. It was made on purpose for me by Bourdon, of Paris, this last summer, and is graduated up to 800 atmospheres.

" When the reaction is finished, the pressure exceeds 500

atmospheres; but it almost immediately falls, stopping at 320 atmospheres. If the screw-tap at the end of the tube be opened at this moment, a jet of liquid may be distinctly seen escaping with extreme violence. If the tap be then closed, a few seconds later a second jet may be obtained, this time less abundant. Pieces of charcoal, slightly kindled, placed in this jet, ignite spontaneously with incredible violence. I have not yet been able to collect the liquid, on account of the force with which it is projected; but I am now endeavoring to combine a cooled gauge, which may, by means of cloths, retain a little of the liquid.

"Yesterday (Monday, December 24th, 1877,) I performed this experiment a second time before a good part of the members of our Physical Society, and we had three well-defined jets, one after the other. I cannot yet give the minimum pressure necessary, for it is evident that I must have had an exaggeration of the pressure produced by an accumulation of the gas in the retort, which resists condensation in the narrow space represented by the inner tube.

"I intend using a similar apparatus to attempt the condensation of hydrogen and nitrogen, and I rely especially on my ability to maintain low temperatures with ease, by steam power.

"I believe that it is in this direction particularly that it is necessary to work to effect the rebellious condensations, since the tension of saturated vapors is a direct function of the temperature. I am having a plan of my apparatus drawn up, and it will be my pleasure and duty to send it to you in the course of the week. I have learned with great interest that M. Pictet has arrived at the same result with myself, and almost at the same moment. I have no idea as to what his process may be, but we shall doubtless enter into correspondence before long, and exchange our ideas on this interesting subject."—*London Pharmaceutical Journal.*

